# Breast cancer molecular diagnostics in Rwanda: a cost-minimization study of immunohistochemistry versus a novel GeneXpert^®^ mRNA expression assay

**DOI:** 10.2471/BLT.22.288800

**Published:** 2022-11-02

**Authors:** Parsa Erfani, Esther Gaga, Emmanuel Hakizimana, Emmanuel Kayitare, Jean Claude Mugunga, Cyprien Shyirambere, Dan A Milner, Lawrence N Shulman, Deogratias Ruhangaza, Temidayo Fadelu

**Affiliations:** a Harvard Medical School, Boston, United States of America (USA).; b University of Global Health Equity, Butaro, Rwanda.; c Ministry of Health, Butaro Hospital, Butaro, Rwanda.; d Partners In Health, Boston, USA.; e Partners In Health/Inshuti Mu Buzima, Butaro, Rwanda.; f American Society for Clinical Pathology, Chicago, USA.; g Abramson Cancer Center, University of Pennsylvania, Philadelphia, USA.; h Dana-Farber Cancer Institute, 450 Brookline Avenue, MA-1B-17, Boston, Massachusetts 02215, USA.

## Abstract

**Objective:**

To compare the financial and time cost of breast cancer biomarker analysis by immunohistochemistry with that by the Xpert^®^ STRAT4 assay.

**Methods:**

We estimated costs (personnel, location, consumables and indirect) and time involved in breast cancer diagnosis at the Butaro Cancer Centre of Excellence, Rwanda, using time-driven activity-based costing. We performed a cost-minimization analysis to compare the cost of biomarker analysis for estrogen receptor, progesterone receptor and human epidermal growth factor receptor-2 status with immunohistochemistry versus STRAT4. We performed sensitivity analyses by altering laboratory-specific parameters for the two methods.

**Findings:**

We estimated that breast cancer diagnosis in Rwanda costs 138.29 United States dollars (US$) per patient when conducting biomarker analysis by immunohistochemistry. At a realistic immunohistochemistry antibody utilization efficiency of 70%, biomarker analysis comprises 48.7% (US$ 67.33) of diagnostic costs and takes 33 min. We determined that biomarker analysis with STRAT4 yields a reduction in diagnosis cost of US$ 7.33 (10.9%; 7.33/67.33), and in pathologist and technician time of 20 min (60.6%; 20/33), per patient. Our sensitivity analysis revealed that no cost savings would be made in laboratories with antibody utilization efficiencies over 90%, or where only estrogen and/or progesterone receptor status are assessed; however, such operational efficiencies are unlikely, and more laboratories are pursuing human epidermal growth factor receptor-2 analysis as targeted therapies become increasingly available.

**Conclusion:**

Breast cancer biomarker analysis with STRAT4 has the potential to reduce the required human and capital resources in sub-Saharan African laboratories, leading to improved treatment selection and better clinical outcomes.

## Résumé

**Objectif:**

Comparer l'analyse des biomarqueurs du cancer du sein par immunohistochimie avec le test Xpert^®^ STRAT4 en termes de coût en temps et en argent.

**Méthodes:**

Nous avons estimé les coûts (personnel, lieu, fournitures et charges indirectes) et le temps requis pour diagnostiquer un cancer du sein au Butaro Cancer Centre of Excellence (Rwanda), à l'aide d'un système de calcul des coûts par activité piloté par le temps (TDABC). Nous avons mené une étude de réduction des coûts afin de comparer les frais engendrés par une analyse des biomarqueurs pour le statut du récepteur d'œstrogène, récepteur de progestérone et récepteur 2 du facteur de croissance épidermique humain, par immunohistochimie d'une part et par STRAT4 d'autre part. Enfin, nous avons effectué des analyses de sensibilité en altérant les paramètres spécifiques aux laboratoires pour les deux méthodes.

**Résultats:**

Nous avons déterminé que le diagnostic du cancer du sein au Rwanda coûtait 138,29 dollars américains (USD) par patient lors d'une analyse des biomarqueurs par immunohistochimie. En tenant compte d'une efficacité d'utilisation réaliste des anticorps en immunohistochimie, fixée à 70%, l'analyse des biomarqueurs représente 48,7% (67,33 USD) des frais engendrés par le diagnostic et dure 33 minutes. De son côté, l'analyse des biomarqueurs par STRAT4 permet de faire baisser les coûts à 7,33 USD (10,9%; 7,33/67,33), tandis que le pathologiste et le technicien n'y consacrent que 20 minutes (60,6%; 20/33) par patient. Notre analyse de sensibilité a révélé qu'aucune économie ne serait réalisée en laboratoire si l'efficacité d'utilisation des anticorps dépasse les 90%, ou si le statut du récepteur d'œstrogène et/ou de progestérone est le seul à être évalué; néanmoins, de tels niveaux d'efficacité opérationnelle sont peu probables, et de plus en plus de laboratoires misent sur l'analyse du récepteur 2 du facteur de croissance épidermique humain vu l'amélioration de l'accessibilité aux thérapies ciblées.

**Conclusion:**

L'analyse des biomarqueurs du cancer du sein par STRAT4 pourrait potentiellement mobiliser moins de ressources humaines et financières dans les laboratoires d'Afrique subsaharienne, entraînant une meilleure sélection des traitements et de meilleurs résultats cliniques.

## Resumen

**Objetivo:**

Comparar el coste económico y de tiempo del análisis de biomarcadores de cáncer de mama mediante inmunohistoquímica con el del ensayo Xpert® STRAT4.

**Métodos:**

Se estimaron los costes (de personal, localización, consumibles e indirectos) y el tiempo que conlleva el diagnóstico del cáncer de mama en el Centro de Excelencia en Cáncer de Butaro (Ruanda), utilizando un cálculo de costes en función del tiempo. También se realizó un análisis de minimización de costes para comparar el coste del análisis de biomarcadores en relación con el estado del receptor de estrógeno, el receptor de progesterona y el receptor 2 del factor de crecimiento epidérmico humano con inmunohistoquímica frente a STRAT4. Asimismo, se realizaron análisis de sensibilidad al modificar los parámetros específicos del laboratorio para los dos métodos.

**Resultados:**

Se ha estimado que el diagnóstico del cáncer de mama en Ruanda cuesta 138,29 dólares estadounidenses (US$) por paciente cuando se realiza el análisis de biomarcadores por inmunohistoquímica. Con una eficiencia de utilización de anticuerpos de inmunohistoquímica realista del 70 %, el análisis de biomarcadores comprende el 48,7 % (US$ 67,33) de los costes para el diagnóstico y tarda 33 min. Se determinó que el análisis de biomarcadores con STRAT4 supone una reducción del coste del diagnóstico de US$ 7,33 (10,9 %; 7,33/67,33), y del tiempo del patólogo y del técnico de 20 min (60,6 %; 20/33), por paciente. El análisis de sensibilidad reveló que no se produciría ningún ahorro de costes en los laboratorios con eficiencias de utilización de anticuerpos superiores al 90 %, o en los que solo se evalúa el estado de los receptores de estrógeno o progesterona; sin embargo, es poco probable que se produzcan estas eficiencias operativas, y cada vez son más los laboratorios que realizan el análisis del receptor 2 del factor de crecimiento epidérmico humano a medida que se dispone de más tratamientos dirigidos.

**Conclusión:**

El análisis de biomarcadores del cáncer de mama mediante STRAT4 tiene el potencial de reducir los recursos humanos y de capital requeridos en los laboratorios del África subsahariana, lo que conduce a una mejor selección del tratamiento y a mejores desenlaces clínicos.

## ملخص

الغرض مقارنة التكلفة المالية والتكلفة الزمنية لتحليل المحددات الحيوية لسرطان الثدي عن طريق الكيمياء الهيستولوجية المناعية، مع تلك الخاصة بفحص Xpert® STRAT4.

الطريقة قمنا بتقدير التكاليف (الخاصة بفريق العمل، والموقع، والمواد الاستهلاكية وغير المباشرة) والوقت المستغرق في تشخيص سرطان الثدي في مركز Centre of Excellence للسرطان في بوتارو برواندا، باستخدام التكلفة المعتمدة على النشاط والوقت. أجرينا تحليلًا لتقليل التكلفة لمقارنة تكلفة تحليل المحددات الحيوية لمستقبلات هرمون الاستروجين، ومستقبلات البروجسترون، وحالة مستقبلات 2 لعامل نمو البشرة البشري مع الكيمياء الهيستولوجية المناعية مقابل STRAT4. أجرينا تحليلات الحساسية عن طريق تغيير المعايير الخاصة بالمختبر للطريقتين.

النتائج لقد قدرنا أن تشخيص سرطان الثدي في رواندا يكلف 138.29 دولارًا أمريكيًا ($US) لكل مريض عند إجراء تحليل المحددات الحيوية بواسطة الكيمياء الهيستولوجية المناعية. عند مستوى كفاءة الاستخدام الواقعي للأجسام المضادة للكيمياء الهيستولوجية المناعية بنسبة %70، يشتمل تحليل المحددات الحيوية على %48.7 (67.33 دولارًا أمريكيًا) من تكاليف التشخيص ويستغرق 33 دقيقة. لقد قررنا أن تحليل المحددات الحيوية باستخدام STRAT4 ينتج عنه انخفاض في تكلفة التشخيص قدره 7.33 دولارًا أمريكيًا (%10.9؛ 7.33/67.33)، وفي وقت أخصائي علم الأمراض والفني 20 دقيقة (%60.6؛ 20/33) لكل مريض. كشف تحليل الحساسية لدينا أنه لن يتم توفير التكاليف في المختبرات ذات كفاءة استخدام الأجسام المضادة التي تزيد عن %90، أو حيث يتم تقييم حالة مستقبلات هرمون الاستروجين و/أو البروجسترون فقط؛ ومع ذلك، فإن مثل هذه الكفاءات التشغيلية غير محتملة، وتتابع المزيد من المختبرات تحليل مستقبلات 2 لعامل نمو البشرة البشري حيث أصبحت العلاجات المستهدفة متاحة بشكل متزايد.

الاستنتاج إن تحليل المحددات الحيوية لسرطان الثدي باستخدام STRAT4 لديه القدرة على تقليل الموارد البشرية والمالية المطلوبة في مختبرات جنوب الصحراء الكبرى في أفريقيا، مما يؤدي إلى اختيار لعلاج مُحسّن ونتائج إكلينيكية أفضل.

## 摘要

**目的:**

旨在比较通过免疫组化法和 Xpert^®^ STRAT4 化验进行乳腺癌生物标志物分析的经济和时间成本。

**方法:**

我们使用时间驱动作业成本法，估计了卢旺达 Butaro 癌症中心在乳腺癌诊断方面投入的成本（人员、场地、消耗品和间接成本）和时间。我们进行了成本最小化分析，比较采用免疫组化与 STRAT4 方法对雌激素受体、孕酮受体和人表皮生长因子受体-2 状态进行生物标志物分析的成本。我们通过改变两种方法的实验室特定参数进行敏感性分析。

**结果:**

我们通过免疫组化法进行生物标志物分析，估计出卢旺达每位患者的乳腺癌诊断成本为 138.29 美元 (US$)。在实际免疫组化抗体利用率为 70% 的情况下，生物标志物分析占诊断成本的 48.7%（67.33 美元），耗时 33 分钟。我们确定，使用 STRAT4 进行生物标志物分析可减少 7.33 美元的诊断成本（10.9%；7.33/67.33），病理学家和技师在每位患者身上花费的时间为 20 分钟（60.6%；20/33）。我们的敏感性分析显示，在抗体利用率超过 90% 的实验室，或只评估雌激素和/或孕酮激素受体状态的实验室，没有成本节约；然而，这样的操作效率是不可能实现的，随着靶向疗法越来越多，越来越多的实验室正在开展人类表皮生长因子受体-2 的分析。

**结论:**

使用 STRAT4 乳腺癌生物标志物分析可能会减少撒哈拉以南非洲地区实验室所需的人力和财力资源，从而改善治疗选择和获得更好的临床结果。

## Резюме

**Цель:**

Сравнить финансовые и временные затраты на анализ биомаркеров рака молочной железы для метода иммуногистохимии и анализа Xpert^®^ STRAT4**.**

**Методы:**

Проведена оценка затрат (персонал, местоположение, расходные материалы и косвенные затраты) и времени, затраченного на диагностику рака молочной железы в Онкологическом центре передовых технологий в Бутаро, Руанда, с использованием калькуляции затрат по видам деятельности. Для сравнения стоимости анализа биомаркеров рецепторов эстрогенов, рецепторов прогестерона и рецептора человеческого эпидермального фактора роста 2-го типа методом иммуногистохимии по сравнению со STRAT4 был проведен анализ минимизации затрат. Также был проведен анализ чувствительности путем изменения специфичных для лаборатории параметров двух методов.

**Результаты:**

Согласно проведенным расчетам, диагностика рака молочной железы в Руанде обходится в 138,29 доллара США на пациентку при проведении анализа биомаркеров методом иммуногистохимического исследования. При реалистичной эффективности использования антител для иммуногистохимического исследования на уровне 70% анализ биомаркеров составляет 48,7% (67,33 доллара США) от стоимости диагностики и занимает 33 минуты. Установлено, что анализ биомаркеров с использованием STRAT4 позволяет снизить стоимость диагностики для одной пациентки на 7,33 доллара США (10,9%; 7,33/67,33), а время работы патолога и техника – на 20 минут (60,6; 20/33). Результаты проведенного анализа чувствительности показали, что в лабораториях с эффективностью использования антител более 90% или при оценке статуса рецепторов только эстрогенов и (или) прогестеронов экономии затрат не ожидается, однако такая эффективность работы маловероятна, и все больше лабораторий проводят анализ рецепторов человеческого эпидермального фактора роста 2-го типа, поскольку таргетная терапия становится все более доступной.

**Вывод:**

Анализ биомаркеров рака молочной железы с использованием STRAT4 способен сократить необходимые человеческие и финансовые ресурсы в лабораториях стран Африки к югу от Сахары, что приведет к более эффективному выбору лечения и улучшению клинических результатов.

## Introduction

Breast cancer is the most common cancer worldwide and the leading cause of cancer death among women.^1^ With ongoing demographic transitions, breast cancer incidence and mortality rates are rising in low- and middle-income countries, especially in sub-Saharan Africa.^1^ To combat the persistent global inequity in breast cancer mortality, the World Health Organization (WHO) recently launched the Global Breast Cancer Initiative, which identifies timely and accurate breast cancer diagnostics as one of its three central pillars.^1^ The WHO Science Council has also recommended avenues to increase access to molecular and genomic diagnostic technologies in low- and middle-income countries.^2^

The breast cancer diagnostic pathway includes tissue biopsy, tissue processing, histopathology and, if histopathology reveals invasive cancer, biomarker analysis for three breast cancer biomarkers, including estrogen receptor, progesterone receptor and human epidermal growth factor receptor-2 status. Biomarker analysis – routinely performed by immunohistochemistry – is essential for staging and systemic therapy selection. Patients with non-metastatic breast cancer positive for estrogen receptor and/or progesterone receptor have a 50% reduced recurrence risk with five years of adjuvant endocrine therapy, which is widely available as an oral medication in low- and middle-income countries.^3^ Similarly, patients with non-metastatic breast cancer positive for human epidermal growth factor receptor-2 have a greater than 40% reduction in recurrence risk with targeted therapies such as trastuzumab or biosimilars, which are becoming increasingly available in low- and middle-income country markets.^4^^,^^5^ Tailored use of endocrine therapies and therapies targeting human epidermal growth factor receptor-2 also yield significant survival benefits for eligible patients with metastatic breast cancer.^6^

However, limited access to reliable immunohistochemistry for biomarker analysis in sub-Saharan Africa represents a major gap in breast cancer care, contributing to poor breast cancer survival in the region.^7^ Immunohistochemistry is a technically complex process that requires robust supply chains, extensive personnel training and continued quality control;^8^ recent surveys across sub-Saharan Africa report that only 42–50% of centres that process breast tissue perform biomarker analysis.^9^^,^^10^ Of the centres that do perform immunohistochemistry, many struggle to maintain consistent services because of a lack of local suppliers and/or regular stock-outs of reagents (often for months).^10^

Innovations in molecular diagnostics may help overcome barriers to breast cancer biomarker analysis. One such example is the Xpert® STRAT4 Assay, a near point-of-care molecular diagnostic technology that runs on the GeneXpert® platform technology. STRAT4 quantitates the mRNAs (messenger ribonucleic acid) for *ESR1*, *PGR*, *ERBB2* and *MKi67* (a proliferation marker) in a closed-system, fully standardized cartridge using reverse transcription polymerase chain reaction.^11^ The assay has shown high concordance with immunochemistry and fluorescence in situ hybridization for *ESR1*-estrogen receptor, *PGR*-progesterone receptor and *ERBB2-*human epidermal growth factor receptor-2, with overall percentage agreements of 98, 90 and 93%, respectively.^12^^–^^14^ A recent study in Rwanda was the first to show immunohistochemistry–STRAT4 concordance in sub-Saharan Africa, with an overall percentage agreement of 93% for *ESR1*-estrogen receptor and 98% for *ERBB2*-human epidermal growth factor receptor-2.^11^

STRAT4 is especially attractive for low-resource settings because most of its reagents are available in a single kit, and its simple manual steps and internal quality controls overcome the need for extensive personnel training. Furthermore, GeneXpert® is a cross-cutting platform that is widely disseminated across Africa and is used across several disease processes, including tuberculosis and human immunodeficiency virus detection.^11^ Although the adoption of STRAT4 could streamline and increase access to breast cancer molecular diagnostics in sub-Saharan Africa, little is known about the real-world impact of STRAT4 implementation in low- and middle-income countries, including its financial and time cost.^15^ Moreover, robust costing data for breast cancer diagnosis are lacking in sub-Saharan Africa, including for biomarker analysis; in a recent scoping review of breast cancer costing studies in low- and middle-income countries, only two studies were identified from sub-Saharan Africa.^16^ These studies (from Kenya and Nigeria) only reported costs of histopathology, but not biomarker analysis.^17^^,^^18^ A cost comparison of breast cancer biomarker analysis with STRAT4 and with immunochemistry could help to quantify the impact of STRAT4 adoption on hospital and government budgets.

The objectives of our study were therefore to: (i) estimate the cost of the breast cancer diagnostic pathway at a low-resource hospital in sub-Saharan Africa using a micro-costing approach; (ii) identify the main drivers of breast cancer diagnosis costs, including the role of biomarker analysis; and (iii) conduct a cost-minimization analysis of breast cancer biomarker analysis by comparing immunohistochemistry with STRAT4.

## Methods

### Study setting and design

We conducted a cost-minimization study to compare the cost of breast cancer diagnosis, including biomarker analysis, using immunohistochemistry and STRAT4 at the Butaro Cancer Centre of Excellence in Rwanda.^19^ The laboratory provides comprehensive breast cancer diagnosis, including tissue processing, histological diagnosis and biomarker analysis for estrogen receptor, progesterone receptor and human epidermal growth factor receptor-2 status with manual immunohistochemistry on formalin-fixed paraffin-embedded samples. As part of an immunohistochemistry–STRAT4 concordance study, we also performed biomarker analysis with STRAT4 for select formalin-fixed paraffin-embedded samples.^11^ We estimated costs using a health-care perspective with time-driven activity-based costing, a micro-costing approach that estimates the costs of health-care resources consumed as a patient moves along a care process (referred to as a process map).^20^ This method was used to quantify differences in cost, time, personnel and consumables required for the two methods of biomarker analysis.

### Process map

We developed the breast cancer diagnosis process map, starting with a patient’s initial visit for breast mass evaluation and ending with the delivery of a breast cancer diagnosis result to the patient, from interviews with involved personnel and shadowing of patients. We interviewed 10 personnel (a pathologist, an oncologist, two internists, a general practitioner, two nurses, two laboratory technicians and a pharmacist) using a semi-structured questionnaire (data repository).^21^ We followed patients until no new process map branches were identified (*n* = 40 patients; 20 patients over days 1 and 3 combined, and a further 20 patients during day 2). For non-patient care processes (laboratory steps), we followed breast tissue samples throughout the laboratory process; we shadowed laboratory technicians twice for standard tissue fixation, processing, histopathology and biomarker analysis with immunohistochemistry and STRAT4. We recorded time estimates during shadowing, and extracted laboratory repeat rates and batching estimates from personnel interviews.

### Costing analysis

We included the cost categories of personnel, location, consumables and indirect costs in estimating the cost of the breast cancer diagnosis pathway. After recording costs in Rwandan francs (₣), we report these in 2021 United States dollars (US$) using the conversion rate of 990.9 Rwandan ₣ = US$ 1.^22^^,^^23^ We rounded final cost and time estimates to the nearest cent and minute, respectively. We report costs according to the Consolidated Health Economic Evaluation Reporting Standards (data repository),^21^^,^^24^ and provide cost calculations in an Excel calculator (data repository).^21^

#### Personnel and location

To estimate personnel and location cost for each process step, we multiplied the cost per minute (or capacity cost rate) of personnel and location by the probability-weighted time of personnel and location involvement, respectively. Personnel capacity cost rate is defined as the annual salary and benefits of personnel divided by the total minutes of availability per year. Location capacity cost rate is defined as the cost of equipment, electricity per metre squared and construction per metre squared (accounting for annual depreciation) divided by the total minutes of availability (when staffed and operational) of the location per year. Given the equipment inventory and energy needs of the laboratory, we assumed the laboratory used 25% of the total electricity consumption of the hospital.^25^^,^^26^ For laboratory equipment costing more than US$ 3000, we calculated a separate cost capacity rate per item (accounting for annual depreciation and maintenance costs) and multiplied it by the probability-weighted time of machine use per sample to estimate the cost of each laboratory machine per sample. We added the calculated laboratory machine cost per sample to the total laboratory location cost. Laboratory steps accounted for the grouping of samples into batches (average number of samples per batch: 40 for immunochemistry, 28 for STRAT4 sample preparation and 4 for STRAT4 cartridge analysis).

#### Consumables and indirect costs

We estimated the costs of consumables by multiplying the unit price per consumable by the probability of patient consumption. The STRAT4 cartridge is not commercially available in the region, but we estimated a price similar to the Xpert® BCR-ABL Ultra cartridge (i.e. US$ 50.00 per cartridge kit) that is available across sub-Saharan Africa for an oncology indication (chronic myelogenous leukaemia). For consumables in which the price of a single unit per patient could not be calculated (such as laboratory reagents), we used the annual ordering frequency and volume of samples requiring the consumable to estimate the consumable cost per patient. We estimated annual sample volumes by performing an inventory of tissue blocks, haematoxylin and eosin slides, and immunohistochemistry slides (including for three breast cancer biomarkers) processed during a 3-month period (October–December 2019). We used annual antibody ordering frequency and breast cancer immunohistochemistry volume to estimate the estrogen receptor antibody utilization efficiency (i.e. percentage of antibody vial used). We estimated indirect costs by dividing overhead hospital costs for outpatient clinics in 2019 by the number of outpatients served at Butaro Hospital that year. We outline all data sources and assumptions in the data repository.^21^

### Sensitivity analyses

We performed two sets of sensitivity analyses, the first of which was to calculate extremity bound estimates for breast cancer diagnostic costs at Butaro Cancer Centre of Excellence. We altered parameters including time estimates from patient shadowing (25th percentile versus 75th percentile), electricity consumption by the laboratory (15% versus 35% hospital electricity costs), and the estimated annual volume of tissue blocks, haematoxylin and eosin slides, and immunohistochemistry slides (+10% versus −10% estimate). Alterations in laboratory volume estimates affect consumable cost and the antibody utilization efficiency of the laboratory. Our second set of sensitivity analyses aimed to make cost estimates generalizable to other laboratories in sub-Saharan Africa. We altered laboratory-specific variables for immunohistochemistry (e.g. batching volumes, number of receptors evaluated and antibody utilization efficiency) and STRAT4 (e.g. batching volumes and estimated cartridge price) individually. 

## Results

### Process map

**Fig. 1:**
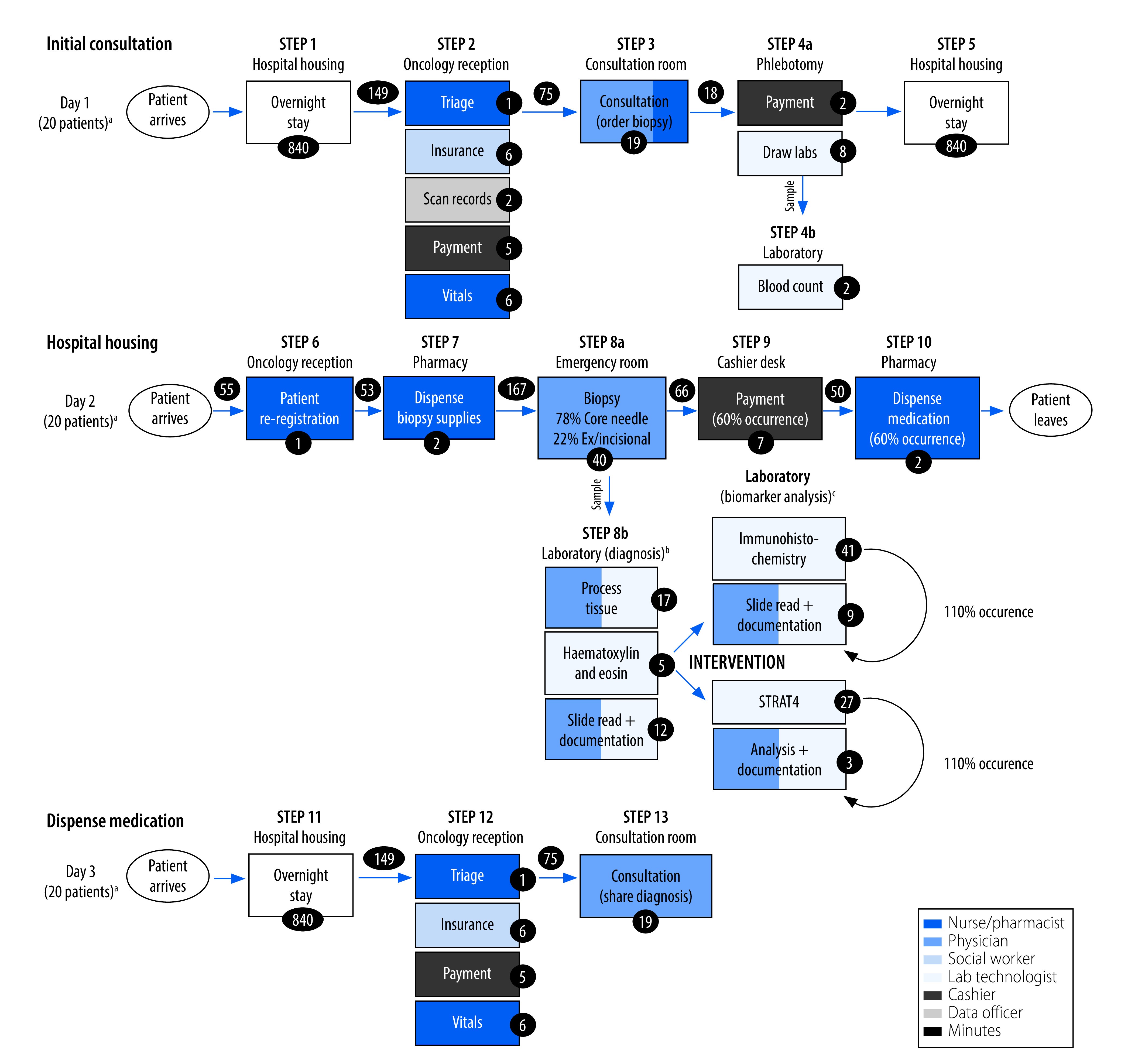
Process map for breast cancer diagnosis pathway, based on personnel interview and patient shadowing at Butaro Cancer Centre of Excellence, Rwanda, 2021

^a^ A total of 40 patients were shadowed: 20 patients were shadowed at the clinic, representing patients presenting for initial consultation (day 1) or follow-up consultation (day 3), and 20 additional patients were shadowed for the biopsy (day 2).

^b^ For Step 8b, Laboratory (diagnosis), time estimates are weighted according to biopsy type.

^c^ All processes in Laboratory (biomarker analysis) have an occurrence of 110% because both biomarker analysis methods had an estimated repeat rate of 10%.

Note: All steps have a 100% probability of occurrence unless otherwise noted.

We identified a 3-day breast cancer diagnosis pathway: initial consultation (day 1), breast mass biopsy (78% by core needle biopsy; 22% by incisional or excisional biopsy) and laboratory analysis (day 2), and follow-up consultation with diagnosis (day 3) (Fig. 1 and data repository).^21^ The laboratory processed 360 breast cancer cases in 1 year, which required 1080 immunohistochemistry stains for the three breast cancer biomarkers. We observed a utilization efficiency of the estrogen receptor antibody of 70%, and assumed antibodies for progesterone receptor and human epidermal growth factor receptor-2 to have the same utilization efficiency. We estimated a laboratory test repeat rate for immunohistochemistry and STRAT4 of 10%.

### Cost of immunohistochemistry

We calculated a cost of breast cancer diagnosis using immunohistochemistry of US$ 138.29 (lower–upper bound estimate: US$ 126.98–149.41; Table 1). Laboratory steps comprised 58.5% (80.88/138.29) of costs: 9.8% (US$ 13.55) for histological diagnosis and 48.7% (US$ 67.33) for biomarker analysis. Consumables were the primary cost drivers, comprising 72.8% (US$ 100.72) of costs. Core needle biopsy was the most expensive consumable, costing US$ 25.35 per patient (data repository).^21^ Personnel costs contributed 18.0% (US$ 24.85) to costs (Table 1), of which physician specialists, the most expensive, comprised 57.8% (US$ 14.37) of personnel costs (data repository).^21^

**Table 1:** Cost of breast cancer diagnosis pathway with immunohistochemistry, Rwanda, 2021

Type of cost	Cost, US$^a^			
	Step along breast cancer diagnosis pathway			Type-of-cost total (% of absolute total) [lower–upper bound estimate]^c^
	Non-laboratory	Laboratory: histologic diagnosis	Laboratory: biomarker analysis^b^	
Personnel	18.25	3.33	3.27	24.85 (18.0) [20.67 to 27.74]
Location^d^	1.42	3.04	2.39	6.85 (5.0) [5.73 to 7.98]
Consumables	31.87	7.18	61.67	100.72 (72.8) [94.72 to 107.83]
Indirect^e^	ND	ND	ND	5.86 (4.2) [NA]
Step cost (% of absolute total)^f^ [lower–upper bound estimate]^c^	51.54 (37.3) [47.22 to 54.58]	13.55 (9.8) [12.76 to 14.42]	67.33 (48.7) [61.13 to 74.55]	138.29 (100) [126.98 to 149.41]

NA: not applicable; ND: not defined; US$: United States dollars.

^a^ Cost in US$ converted from Rwandan francs in 2021. Time and cost data rounded to nearest minute and cent, respectively, for inclusion in table; totals calculated using unrounded data (see data repository).^21^

^b^ Biomarker analysis at Butaro Cancer Centre of Excellence includes manual immunohistochemistry for estrogen receptor, progesterone receptor and human epidermal growth factor receptor-2 status.

^c^ Lower bound estimates include 25th percentile time estimates (non-laboratory steps), 15% of electricity costs attributed to laboratory and estimated annual laboratory volume plus 10% (altering the cost of consumables per patient). Upper bound estimates include 75th percentile time estimates (non-laboratory steps), 35% of electricity costs attributed to laboratory and estimated annual laboratory volume minus 10% (altering the cost of consumables per patient).

^d^ For laboratory equipment costing more than US$ 3000, a separate capacity cost rate was calculated per item of equipment. Based on the length of equipment utilization per sample, an allocated capacity cost rate per item of equipment was added to the location cost.

^e^ Indirect costs include fuel, telephone, internet, security services, cleaning services, office supplies, maintenance, patient food and patient housing.

^f^ Percentage of total costs for non-laboratory and laboratory steps do not add up to 100% because indirect costs (4.2%) are not assigned to any individual step.

### Immunohistochemistry versus STRAT4

We calculated that biomarker analysis by STRAT4 cost US$ 7.33 (lower–upper bound estimate: US$ 1.52 to 14.15) or 10.9% (7.33/67.33) less per patient than for biomarker analysis by immunohistochemistry (Table 2). We noted that the decrease in cost of consumables (US$ 57.38 for STRAT4 versus US$ 61.67 for immunohistochemistry; difference US$ 4.29) contributed the largest portion (58.6%; 4.29/7.33) of the cost saving of STRAT4. Consumables comprised 91.6% (61.67/67.33) of immunohistochemistry costs and 95.6% (57.38/60.00) of STRAT4 costs; biomarker analysis by immunohistochemistry requires 17 consumables, while biomarker analysis by STRAT4 requires only four. For immunohistochemistry, 76.2% (46.97/61.67) of consumable costs were from monoclonal antibodies for estrogen receptor, progesterone receptor and human epidermal growth factor receptor-2 (US$ 12.97, 10.27 and 9.58, respectively, per patient), and for the Dako REAL EnVision Detection System (US$ 14.15 per patient). For STRAT4, 98.8% (56.71/57.38) of consumable costs were from the STRAT4 cartridge kit (US$ 56.71 per patient; data repository).^21^

**Table 2:** Comparison of time and cost for breast cancer biomarker analysis by immunohistochemistry and by Xpert^®^ STRAT4 assay, Rwanda, 2021

Type of cost	Immunohistochemistry			Xpert^®^ STRAT4			Absolute saving (relative saving, %) achieved by using STRAT4	
	Time, minutes^a^	Cost, US$^a^		Time, minutes^a^	Cost, US$^a^		Time, minutes^a^	Cost, US$^a^
Personnel	33	3.27		13	0.91		20 (60.6)	2.37 (72.5)
Location^b^	54	2.39		33	1.72		21 (38.9)	0.67 (28.0)
Consumables^c^	NA	61.67		NA	57.38		NA	4.29 (7.0)
Biomarker analysis [lower–upper bound estimate]^d^	NA	67.33 [61.13 to 74.55]		NA	60.00 [59.61 to 60.40]		NA	7.33 (10.9) [1.52 to 14.15]
Total annual saving [lower–upper bound estimate]^d,e^	NA	NA		NA	NA		14 910	2638.53 [603.22 to 4585.58]

NA: not applicable; US$: United States dollars.

^a^ Cost in US$ converted from Rwandan francs in 2021. Time and cost data rounded to nearest minute and cent, respectively, for inclusion in table; totals calculated using unrounded data (see data repository).^21^

^b^ For laboratory equipment costing more than US$ 3000, a separate capacity cost rate was calculated per item of equipment. Based on the length of equipment utilization per sample, an allocated cost capacity rate per item of equipment was added to the location cost.

^c^ Immunohistochemistry requires 17 consumables; STRAT4 requires 4 consumables. The antibody utilization efficiency for estrogen receptor was 70%; progesterone receptor and human epidermal growth factor receptor-2 antibodies were assumed to have the same utilization efficiency. The price of Xpert® STRAT4 was assumed to be similar to that of the Xpert® BCR-ABL Ultra cartridge at US$ 50 per cartridge kit.

^d^ Lower bound estimates include 15% of electricity costs attributed to laboratory, estimated annual laboratory volume plus 10% (altering cost of consumables per patient); upper bound estimates include 35% of electricity costs attributed to laboratory, estimated annual laboratory volume minus 10% (altering cost of consumables per patient).

^e^ Butaro Cancer Centre of Excellence laboratory assesses 360 breast samples for estrogen receptor, progesterone receptor and human epidermal growth factor receptor-2 annually.

Biomarker analysis using STRAT4 saved 20 min or 60.6% (20/33) of personnel time per patient compared with immunohistochemistry (13 min for STRAT4 versus 33 min for immunohistochemistry; Table 2). The decreased personnel time corresponded to US$ 2.37 in cost savings, which accounted for 32.3% (2.37/7.33) of the savings made by using STRAT4. Of the 20 min saved with STRAT4 (note that data are subject to rounding errors), 15 min were saved by the laboratory technician (11 min for STRAT4 versus 26 min for immunohistochemistry) and 6 min were saved by the pathologist (1 min for STRAT4 versus 7 min for immunohistochemistry; data repository).^21^ With an annual volume of 360 breast samples at the laboratory for biomarker analysis, using STRAT4 instead of immunohistochemistry could save US$ 2638.53 (lower–upper bound estimate: US$ 603.22 to 4585.58) and 122.6 hours of personnel time annually (note that data in Table 2 are rounded to the nearest minute).

### Sensitivity analysis

We did not observe any major effect on cost or personnel time when increasing batch volumes of immunohistochemistry and STRAT4 analyses (Table 3). However, the antibody utilization efficiency of immunohistochemistry impacted the cost savings made by using STRAT4: the cost of biomarker analysis by STRAT4 is almost the same as by immunohistochemistry for laboratories that operate at 90% antibody utilization efficiency (difference: US$ 0.07). We noted that the number of receptors assessed substantially affects the cost comparison: biomarker analysis by STRAT4 costs US$ 38.23 (175.6%) or 13.76 (29.8%) more per patient than by immunohistochemistry at laboratories that only assess estrogen receptor status or estrogen and progesterone receptor status (relative to assessing three breast cancer biomarkers), respectively. For laboratories that assess the status of proliferation marker Ki67 (in addition to the other three biomarkers), the use of STRAT4 results in a saving of US$ 22.78 (27.5%) per patient. We also observed that the cost of the cartridge is important; if the STRAT4 cartridge is made available at the same price as the Xpert® severe acute respiratory syndrome coronavirus 2 (US$ 14.90 per kit) or the *Mycobacterium tuberculosis*/rifampin resistance Ultra (US$ 9.98 per kit) cartridges, the use of STRAT4 instead of immunohistochemistry would result in a cost saving of US$ 45.94 (68.2%) or US$ 51.35 (76.3%) per patient, respectively.

**Table 3:** Sensitivity analyses of laboratory-specific parameters to compare time and cost estimates of breast cancer biomarker analysis using immunohistochemistry and Xpert^®^ STRAT4 assay, Rwanda, 2021

Parameter	Immunohistochemistry			Xpert^®^ STRAT4			Absolute saving (relative saving, %) achieved by using STRAT4	
	Personnel time, minutes	Cost, US$		Personnel time, minutes	Cost, US$		Personnel time, minutes	Cost, US$
**Cost estimate per patient^a^**	33	67.33		13	60.00		20 (60.6)	7.33 (10.9)
**Immunohistochemistry batches (primary: 40 slides)**								
72 slides	31	66.80		13	60.00		18 (58.1)	6.80 (10.2)
20 slides	38	68.53		13	60.00		25 (65.8)	8.53 (12.4)
**Antibody utilization efficiency (primary: 70%)^b^**								
90% of antibody vial used	33	60.07		13	60.00		20 (60.6)	0.07 (0.1)
80% of antibody vial used	33	63.27		13	60.00		20 (60.6)	3.27 (5.2)
60% of antibody vial used	33	72.85		13	60.00		20 (60.6)	12.85 (17.6)
**No. receptors analysed (primary: three)**								
One (estrogen receptor)	11	21.77		13	60.00		−2 (−18.2)	−38.23 (−175.6)
Two (estrogen and progesterone receptors)	22	46.24		13	60.00		9 (40.9)	−13.76 (−29.8)
Four (estrogen and progesterone receptors, human epidermal growth factor receptor-2, Ki67)	44	82.78		13	60.00		31 (70.5)	22.78 (27.5)
**STRAT4 batching (primary: 4-module GeneXpert^®^ system)^c^**								
16-module GeneXpert^®^ system	33	67.33		13	59.33		20 (60.6)	8.00 (11.9)
2-module GeneXpert^®^ system	33	67.33		13	60.98		20 (60.6)	6.35 (9.4)
**Xpert® cartridge price (primary: US$ 50.00)^d^**								
US$ 40.00	33	67.33		13	49.00		20 (60.6)	18.33 (27.2)
US$ 30.00	33	67.33		13	38.00		20 (60.6)	29.33 (43.6)
US$ 20.00	33	67.33		13	27.00		20 (60.6)	40.33 (59.9)
US$ 14.90 (price of Xpert^®^ SARS-CoV-2 cartridge kit)	33	67.33		13	21.39		20 (60.6)	45.94 (68.2)
US$ 9.98 (price of Xpert^®^ MTB/RIF Ultra cartridge kit)	33	67.33		13	15.98		20 (60.6)	51.35 (76.3)

MTB/RIF: *Mycobacterium tuberculosis*/rifampin resistance; SARS-CoV-2: severe acute respiratory syndrome coronavirus 2; US$: United States dollars.

^a^ Estimates are taken from Table 2.

^b^ The antibody utilization efficiency was based on the estrogen receptor antibody utilization efficiency of the laboratory.

^c^ 2-, 4- and 16-module batching for STRAT4 corresponds to 14-, 28- and 112-sample batching for preparation, respectively, assuming that the GeneXpert® system can be run seven times in 1 day (each run takes 70 min).

^d^ The price of the 2-, 4- and 16-module Xpert® machines was US$ 12 030, 17 500 and 64 350, respectively. The price of Xpert® STRAT4 was assumed to be similar to that of the Xpert® BCR-ABL Ultra cartridge, that is, US$ 50 per cartridge kit.

Note: Cost in US$ converted from Rwandan francs in 2021. Time and cost data rounded to nearest minute and cent, respectively, for inclusion in table.

## Discussion

For a real-world immunohistochemistry antibody utilization efficiency of 70%, we demonstrated that STRAT4 is a moderate cost saving and robust time-saving alternative to immunochemistry for biomarker analysis of three breast cancer biomarkers. However, our sensitivity analysis showed that when laboratories operate at antibody utilization efficiencies of 90% or greater, STRAT4 is more expensive than immunohistochemistry. Although there are no reports in the literature of antibody utilization efficiencies for laboratories in sub-Saharan Africa, operational and supply-chain challenges likely prevent them from achieving 90% efficiency, as was the case at Butaro Cancer Centre of Excellence (which has an antibody utilization efficiency of 70%).^10^ Less-specialized laboratories may operate at even lower immunohistochemistry efficiencies, meaning that STRAT4 could yield even greater cost and time savings for these facilities. 

We also demonstrated that the cost savings of STRAT4 were also sensitive to the number of breast cancer molecular markers assessed. While all four biomarkers (*ESR1, PGR, ERBB2* and *MKi67*) reported with the STRAT4 assay are clinically meaningful, their results are not always actionable in low- and middle-income countries. STRAT4 has fixed costs irrespective of the intended number of biomarkers, and may cost more for cancer programmes that are currently performing immunohistochemistry only for hormone receptor status (estrogen and/or progesterone). However, as therapies targeting human epidermal growth factor receptor-2 (including biosimilars) become increasingly available, more laboratories in sub-Saharan Africa (including Butaro Cancer Centre of Excellence) are pursuing routine human epidermal growth factor receptor-2 analysis.^5^

Given the expanding footprint of GeneXpert® across Africa, there is growing interest in the potential of STRAT4 to streamline and decentralize breast cancer molecular diagnostics to less-specialized facilities. Many laboratories across sub-Saharan Africa have the capability to process tissue and diagnose breast cancer, but do not perform immunohistochemistry because of financial, technical and supply-chain limitations.^9^^,^^10^ Our results suggest that STRAT4 has the potential to reduce the human and capital resources necessary for these laboratories to perform breast cancer biomarker analysis. Given the scarcity of pathologists and laboratory technicians in sub-Saharan Africa, the time saved by personnel with STRAT4 is incredibly valuable and can enable personnel to complete additional laboratory processes.^10^^,^^27^ Preliminary data also suggest that the STRAT4 assay has similarly favourable diagnostic performance with fine-needle aspirate samples, yielding the potential to expand availability of breast cancer molecular diagnostics to smaller district-level hospitals where traditional biopsies and tissue processors are not available.^28^

Although STRAT4 may reduce human and capital barriers for breast cancer biomarker analysis, its absolute cost of US$ 60 will likely limit its wide adoption in sub-Saharan Africa (where the annual health-care spending per capita is US$ 84).^29^ The primary cost of diagnostics with GeneXpert® is the price of the cartridge. In the past decade, global multisectoral investments have helped to lower the price of the Xpert® tuberculosis diagnostic assays to approximately US$ 10 per cartridge, transforming tuberculosis care in sub-Saharan Africa.^30^ Driving down the price for STRAT4 could similarly revolutionize access to breast cancer molecular analysis.

The adoption of STRAT4 instead of breast immunohistochemistry by laboratories will likely be influenced by several implementation factors beyond cost, such as assay training, turn-around time and supply-chain feasibility. First, in our experience, training for STRAT4 (conducted over 2 days via video teleconference) was substantially less burdensome than that for manual immunohistochemistry, which required extensive and repeated in-person trainings to maintain reproducible results.^31^ Facilities with fewer skilled personnel may therefore be more willing to adopt STRAT4. Second, given that STRAT4 requires substantially less personnel time, it may also decrease turn-around time for breast cancer diagnostic results (currently about 2 weeks at Butaro Cancer Centre of Excellence), which is critical for initiating optimal therapy. Finally, given that STRAT4 requires substantially fewer consumables, it may be less vulnerable to supply-chain challenges. The 17 reagents required for immunohistochemistry rely on complex supply chains; when one reagent is out of stock, the entire process is halted, leading to diagnostic delays (a common issue for immunohistochemistry implementation in sub-Saharan Africa).^10^ Although GeneXpert® testing relies on fewer reagents, its use in sub-Saharan Africa has also been limited by stock-outs and non-functional modules.^32^

Our study has several limitations. First, this cost-minimization analysis assumes that the diagnostic outcomes for immunohistochemistry and STRAT4 are equivalent: a reasonable assumption for laboratories with high-quality immunohistochemistry and in turn high concordance with STRAT4.^11^^,^^14^ For laboratories that struggle to maintain high-quality immunohistochemistry, STRAT4 may be more accurate; a cost–effectiveness analysis is therefore needed to account for downstream cost implications of inaccurate immunohistochemistry results. Second, we did not consider the cost of performing fluorescence in situ hybridization for immunohistochemistry-equivocal cases of human epidermal growth factor receptor-2, because it is not currently available in Rwanda.^11^ If potential fluorescence in situ hybridization costs were added to immunohistochemistry costs, STRAT4 would yield even greater cost savings. Third, training costs for both methods of biomarker analysis were not included in this cost analysis. Given that the cost of biomarker analysis was primarily driven by consumables, we estimate that training cost differences between the two methods may not impact long-term costs but would likely impact upfront costs. Fourth, estimates for laboratory repeat rates and sample batching were drawn from personnel interviews, and therefore vulnerable to recall bias.

Given the challenges of maintaining efficient immunohistochemistry services in low-resource settings such as many sub-Saharan African laboratories, we consider that STRAT4 adoption could reduce the capital and human resource barriers to performing breast cancer biomarker analysis, leading to improved treatment selection and better clinical outcomes.
